# Effects of disparity on visual discomfort caused by short-term stereoscopic viewing based on electroencephalograph analysis

**DOI:** 10.1186/s12938-018-0595-0

**Published:** 2018-11-03

**Authors:** Xiao Wang, Liuye Yao, Yuemei Zhao, Lidong Xing, Zhiyu Qian, Weitao Li, Yamin Yang

**Affiliations:** College of Automation Engineering, Nanjing University of Aeronautics and Astronautics, No. 29 Jiangjun Avenue, Jiangning District, Nanjing, 211106 China

**Keywords:** Stereoscopic depth, Visual discomfort, Visual evoked potential, Directed transfer function/adaptive directed transfer function

## Abstract

**Background:**

Discomfort evoked by stereoscopic depth has been widely concerned. Previous studies have proposed a comfortable disparity range and considered that disparities exceed this range would cause visual discomfort. Brain activity recordings including Electroencephalograph (EEG) monitoring enable better understanding of perceptual and cognitive processes related to stereo depth-induced visual comfort.

**Methods:**

EEG data was collected using a stereo-visual evoked potential (VEP) test system by providing visual stimulus to subjects aged from 21 to 25 with normal stereoscopic vision. For each type of visual stimulus, data were processed using directed transfer function (DTF) and adaptive directed transfer function (ADTF) in combination with subjective feedbacks (comfort or discomfort). The topographies of information flow were constructed to compare responses stimulated by different stereoscopic depth, and to determine the difference in comfort and discomfort situations upon stimulation with same stereoscopic depth.

**Results:**

Based on EEG analysis results, we found that the occipital P270 was moderately related to the disparity. Moreover, the ADTF of P270 showed that the information flows at frontal lobe and central-parietal lobe changed when stimulation with different stereoscopic depth applied. As to the stereo images with same stereoscopic depth, the DTF outflows at the temporal and temporal-parietal lobes in δ band, central and central-parietal lobes in α and θ bands, and the comparison of inflows in these three bands could be considered as discriminated indexes for matching the stereoscopic effect with viewers’ comfort or discomfort state impacted by disparity. The subjective feedbacks indicated that the comfort judgments remained as a result of cumulative effect.

**Conclusions:**

This study proposed a short-term stereo-VEP experiment that shorted the duration of each stimulus in the experimental scheme to minimize the interference from other factors except the disparity. The occipital P270 had a mid-relevance to the disparity and its ADTF showed the affected areas when viewers are receiving stimulations with different disparities. DTF could be considered as discriminated indexes for matching the stereoscopic effect with viewers’ comfort or discomfort state induced by disparity. This study proposed a preferable experiment to observe the single effect of disparity and provided an intuitive and easy-to-read result in a more convenient manner.

## Background

Visual discomfort in stereo viewing is noted as a result of inappropriate binocular disparity. Depth features of stereoscopic images have thus been widely investigated. Lambooij et al. [1] claimed that disparity beyond one degree could cause noticeable visual discomfort. Cho et al. [2] found that the increase of binocular disparity gave rise to human fatigue level. To obtain more in-depth understanding of stereoscopic depth-induced visual discomfort, the relevance between stereo imagery and potential adverse effects have been studied under a wide variety of situations. Shibata et al. [3] reported that large crossed disparity and small uncrossed disparity led to a marked drop in comfort ratings during stereo viewing based on subjective questionnaires. Although such questionnaire-based survey presents a simple and practical method, individual differences in uncertainty tolerance may influence the results. Functional magnetic resonance imaging (fMRI) was also employed to explore the changes in human cortex during visual stereo stimulation study. Based on fMRI, Liu et al. [4] indicated that stereoscopic depth perception was correlated with several regions (hV3A, LG, hMT/V5, LOS and VIPS), which stands for the precise positions on human cortex in stereoscopic vision processing. Jung et al. [5] also used fMRI to locate the activated areas on the cortex when people felt uncomfortable during 3D viewing. fMRI remains a precise yet expensive method which also requires readers to have professional knowledge of the brain structure. For the detection of stereo visual discomfort in daily life and taking the cost-effectiveness into consideration, a relatively simple, easy-operating, labor- and money-saving method needs to be developed. Recently, electroencephalograph (EEG) has been widely used as an effective way to assess stereoscopic visual fatigue. Li et al. [6] found the power of the high frequency band in EEG became stronger in 3D viewing and the peak difference in P700 at 3D oddball paradigm might be an effective indicator for revealing 3D visual fatigue. Mun et al. [7] found significantly reduced P600 amplitudes and delayed P600 latencies appeared in accordance with 3D visual fatigue, and significant fatigue effects were also observed at P4 and O2 sites during the 8.57 Hz attended task. Zou et al. [8] evaluated visual fatigue level via random dot stereogram (RDS) with six different disparities and found that EEG could be employed as a useful tool to predict visual fatigue caused by vergence-accommodation conflict. Malik et al. [9] compared the EEG absolute power differences, coherence and complexity, then concluded that 3D viewing is more attractive than 2D and may cause high attention and involvement of working memory manipulations. Other researches also demonstrated the relevance between visual fatigue and disparity in stereo viewing based on EEG analysis [10–13]. Kang et al. [14] developed a platform to facilitate comfortable stereo video viewing using EEG-based visual discomfort evaluation technology. Frey et al. used EEG and event related potential (ERP) to assess the visual discomfort in stereoscopic displaying and established a prediction model to assess visual discomfort based on their ERP results and reached an accuracy rate more than 62% on average [15, 16]. Although the above work demonstrated the capability of distinguishing uncomfortable stereoscopic viewing conditions from comfortable ones based on EEG analysis, each stimulus in their experimental trial lasted for seconds. In the majority of previous studies, overall level of visual discomfort was recorded according to the subjective questionnaires and was determined after certain experimental session. However, it might be expected that the time course of viewing could also cause possible cumulative effects and thus affect discomfort judgments.

Visual evoked potential (VEP), which refers to the evoked potential caused by a visual stimulus, is commonly used for vision check in clinics. VEP measures the functional integrity of the visual pathways from retina via the optic nerves to the visual cortex when retinas receive stimuli, and can be induced by a stimulus repeated at a higher rate [17]. Therefore, in order to capture the fast responses upon short-term visual stimuli (less than 1 s) with various stereoscopic depth, the present pilot study described an experimental protocol which acquires both EEG signals and subjective feedbacks without interrupting the viewing.

Thanks to precise stereoscopic parameters and real-time measures of our stereo-VEP setup, the effects of disparity and to which degree the disparity would evoke visual discomfort immediately over short viewing sequences were systematically investigated in this study. The effects of disparity over short viewing sequences were systematically investigated by directed transfer function (DTF) and adaptive directed transfer function (ADTF) methods. The study will provide more intuitive and easy-to-read results to the public to help them understand their physical status in a more convenient manner and pave the way for better estimating stereoscopic discomfort and optimization of stereoscopic display parameters in the future.

## Methods

### Experimental protocol

#### Stereo-VEP experimental system

**Fig. 1:**
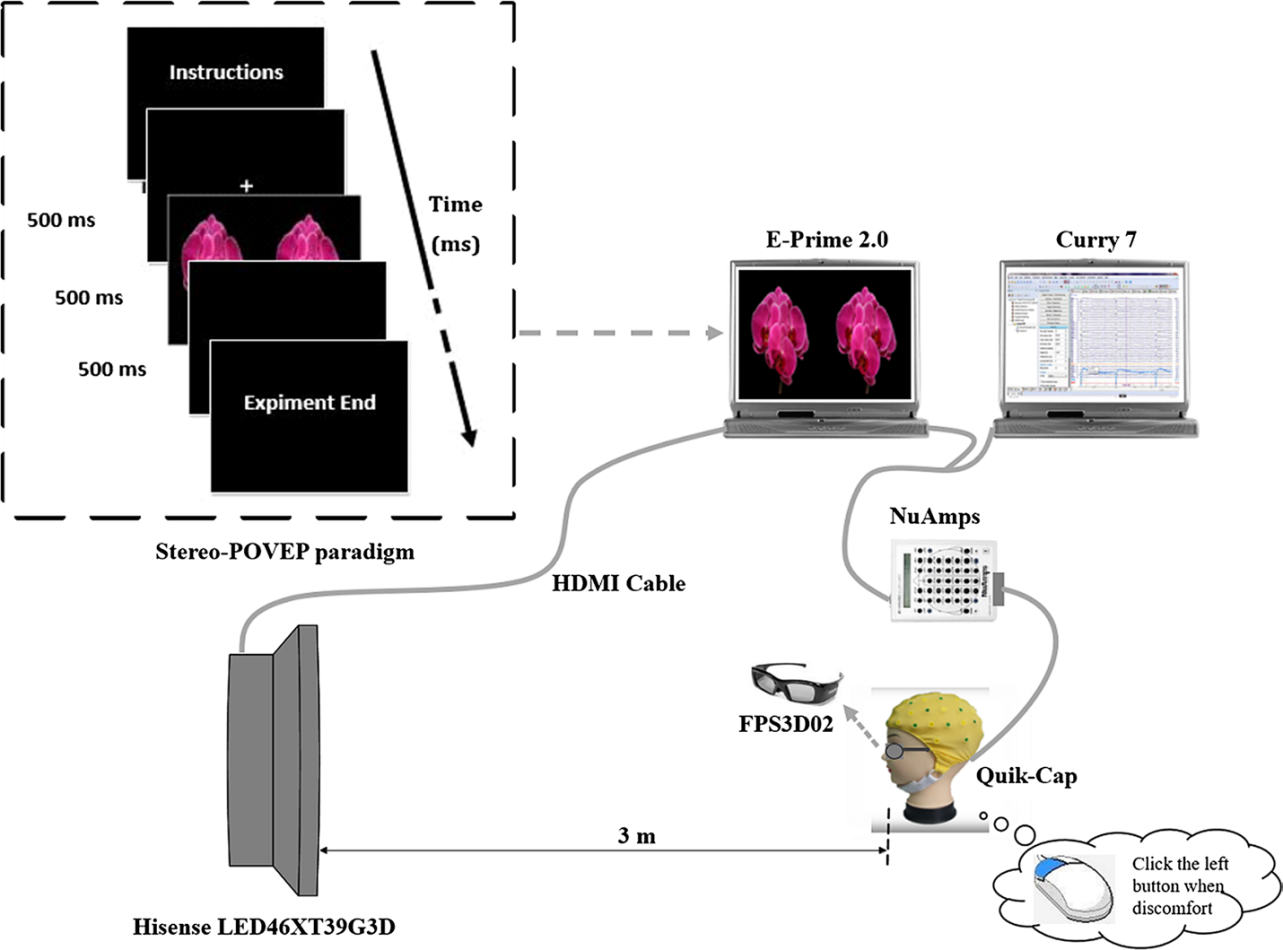
The schematic setup of experimental system

Figure 1 shows the schematic setup of our experimental system which composed of a stimulation part, a recording part and a feedback part. The simulation part is consisted of an active shutter 3D-TV (LED46XT39G3D, Hisense) and a laptop installed with E-Prime 2.0. The visual stimuli were provided by a visual paradigm written based on E-Prime 2.0. During experiment, the paradigm running on the laptop would be synchronously displayed on the 3D-TV through a high definition multimedia interface (HDMI) cable. Participants watched the stereo stimuli series through a pair of shutter glasses (FPS3D02, Hisense). The detailed information of the paradigm will be introduced in the following section.

EEGs were acquired in real-time with NuAmps electroencephalograph (Australia, Neuroscan) during experiment. Signal recording was performed according to the expanded international 10–20 montage system. Thirty of total thirty-seven electrodes were placed along the scalp for EEG recording. As alternative references, two mastoid electrodes (M1 and M2) at bilateral were used during recording. Four electrodes were used for real-time horizontal and vertical electrooculogram (EOG) recording and the ground was set at FPz. The sampling rate was 1 kHz. The contact resistance of each electrode was less than 5 kΩ. The acquisition process was monitored by the other laptop installed with Curry 7. For each trial, participants stimulated upon corresponding visual cues were asked to click the left button of the mouse as soon as they felt uncomfortable.

The entire experiment was conducted in a quiet room and the temperature was kept at 24 °C. Ten right-handed subjects (male: 9, female: 1, aged from 21 to 25) with normal stereoscopic vision participated in our experiment. All subjects were informed about the general experiment information and signed the consent form before the experiment. All experiments were carried out in accordance with institutional guidelines of Nanjing University of Aeronautics and Astronautics (NUAA). All experimental protocols were approved by the Ethics Committee of NUAA.

#### The paradigm

The pattern onset/offset visual evoked potential (POVEP), including both flash visual evoked potential (F-VEP) and pattern visual evoked potential (P-VEP), was applied to explore the relevance between stereoscopic depth perception and human discomfort in this study. The stereo pattern onset/offset visual evoked potential (Stereo-POVEP) paradigm was shown in the dashed box in Fig. 1. After the subject read through the instructions for experiment description, he or she would tap the space key to start the experiment. A cross would appear at the center of the screen for 5 s to draw the participant’s attention to the screen center. One of four images with different disparities would randomly appear and displayed for 500 ms for each, followed by a black background for 500 ms. A complete set of stimulation series presented 240 trials (60 trials for each image stimulus) and it costed less than 5 min in total. Each subject performed two sets of above stimulation trials.

**Fig. 2:**
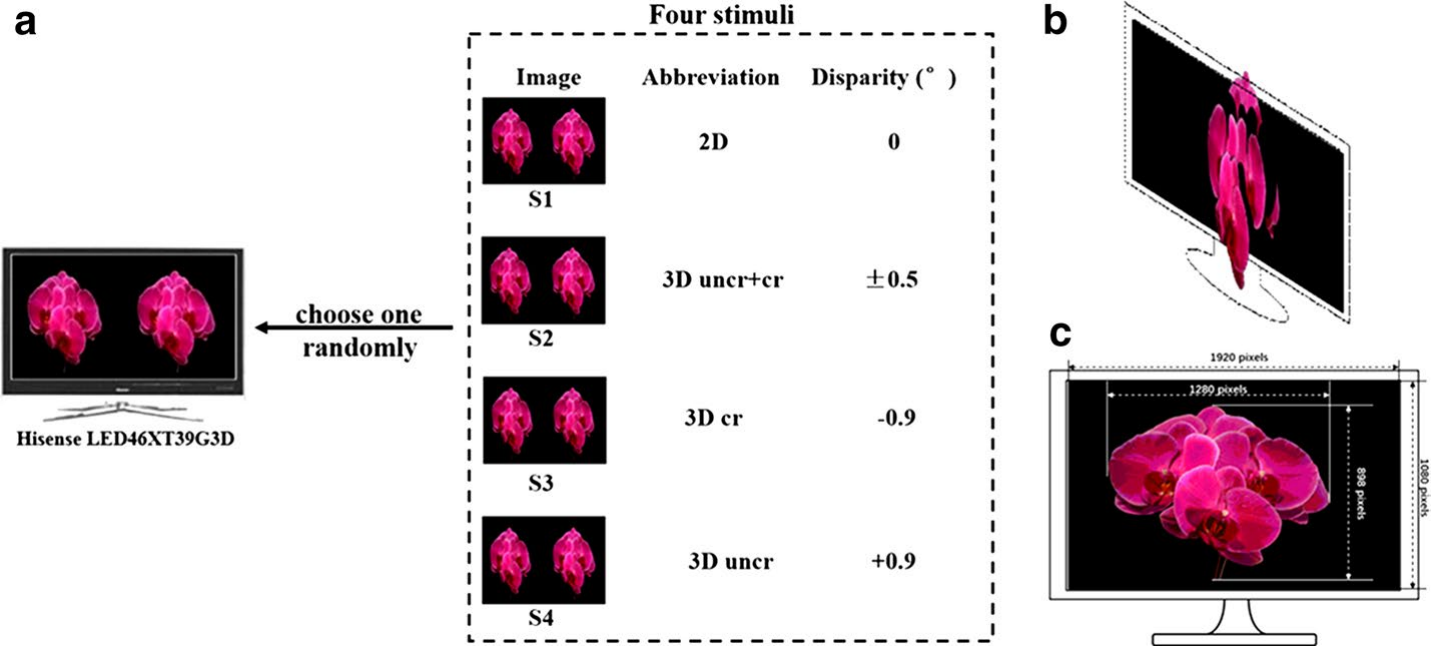
Four image stimuli in the Stereo-POVEP paradigm. **a** The disparity information of four stimuli, respectively, **b** the image stimulus with both uncrossed and crossed disparities (S2), **c** the size information of the image stimuli

The disparity parameters of the stereo images are illustrated in Fig. 2a. The image stimulus with zero disparity includes neither uncrossed disparities nor crossed disparities, and in other words, it is a 2D image stimulus (abbreviated as ‘2D’). The “±” sign in the “disparity” column (abbreviated as ‘3D uncr+cr’) means that, besides the 2D (zero disparity) part of the image, the image stimulus includes uncrossed (orchid bud appeared in front of the screen) and crossed disparities (orchid bud appeared behind the screen) (Fig. 2b). The “+” sign means the uncrossed disparity (abbreviated as ‘3D uncr’), and the “−” sign means the crossed disparity (abbreviated as ‘3D cr’), respectively. For convenience, we numbered these four types of stimuli ‘2D’, ‘3D uncr+cr’, ‘3D cr’ and ‘3D uncr’ as ‘S1’, ‘S2’, ‘S3’ and ‘S4’. ‘S2’ has both negative and positive disparities, and the absolute value of its positive disparity or negative disparity are both in the suggested comfortable range (| positive (or negative) disparity of S2| = 0.5 < 1). The size of the image in left–right format was 1920 × 1080 and the object in the middle of the image was 1280 × 898 (Fig. 2c). The visual angle was approximately 4.08° × 6.67°. All images were provided by Prof. Qiu and his group from the School of Art in Peking University.

#### Data processing

The reference was switched to Cz during offline processing. After baseline correction, 50 Hz notch filtering, 0.01–30 Hz band-pass filtering, eye movement artifacts removal and bad blocks removal, VEPs were averaged by the time-locked and phase-locked EEGs. Valid data used for each averaging was over 90 trials. The processed VEPs were saved for following computations.

### Brain connectivity estimators

#### DTF and ADTF

Granger causality is used to compute the causal relationship between two time series. Kaminski and Blinowska successfully applied DTF into neurobiological system computations, which expanded the application of Granger causality based on multivariate autoregressive (MVAR) model [18]. A short description of this algorithm is as follows.

**X**(t) = [X_1_(t),X_2_(t),X_3_(t),…,X_k_(t)]^T^ is defined as a k-channel signal at time point t. The superscript T represents the matrix transposition. Then the MVAR model can be described as:

1 $$ {\mathbf{X}}\left( {\text{t}} \right) = \sum\limits_{{{\text{n}} = 0}}^{\text{p}} {{\mathbf{A}}\left( {\text{n}} \right){\mathbf{X}}({\text{t}} - {\text{n}}) + {\mathbf{E}}({\text{t}})} $$

with

2 $$ {\mathbf{A}}\left( 0 \right) = {\mathbf{I}} $$

where **E**(t) is the white noise and **A**(1), **A**(2),…,**A**(p) are ${\text{k}} \times {\text{k}}$ coefficient matrices. The order p could be derived from Schwarz Bayesian Criterion (SBC) [19]. Then it is transformed into the frequency domain by Fourier transforms:

3 $$ {\mathbf{A}}\left( {\text{f}} \right){\mathbf{X}}\left( {\text{f}} \right) = {\mathbf{E}}({\text{f}}) $$

where

4 $$ \text{A}\left( \text{f} \right) = \sum\limits_{{{\text{n}} = 0}}^{\text{p}} {{\mathbf{A}}\left( {\text{n}} \right)} {\text{e}}^{{ - 2\uppi{\text{ftn}}}} $$

So **X**(f) could be rewritten as:

5 $$ {\mathbf{X}}\left( {\text{f}} \right) = {\mathbf{A}}^{ - 1} \left( {\text{f}} \right){\mathbf{E}}({\text{f}}) $$

Define **H**(f) = **A**^−1^(f), and then

6 $$ {\mathbf{X}}\left( {\text{f}} \right) = {\mathbf{H}}\left( {\text{f}} \right){\mathbf{E}}({\text{f}}) $$

**H**(f) is the transfer matrix of the system, which equals to the inverse of frequency-transformed coefficient matrix. The casual information from channel j to channel i is:

7 $$ \uptheta_{\text{ij}}^{2} \left( {\text{f}} \right) = \left| {{\mathbf{H}}_{\text{ij}} } \right|^{2} $$

The DTF measurement $\upgamma_{\text{ij}}^{2} \left( {\text{f}} \right)$ is the normalization of $\uptheta_{\text{ij}}^{2} \left( {\text{f}} \right)$, which describes the directional causal information from channel j to channel i. The value of $\upgamma_{\text{ij}}^{2} \left( {\text{f}} \right)$ is between 0 and 1.

8 $$ \upgamma_{\text{ij}}^{2} \left( {\text{f}} \right) = \frac{{\left| {{\mathbf{H}}_{\text{ij}} \left( {\text{f}} \right)} \right|^{2} }}{{\mathop \sum \nolimits_{{{\text{m}} = 1}}^{\text{k}} \left| {{\mathbf{H}}_{\text{im}} \left( {\text{f}} \right)} \right|^{2} }} $$

ADTF was proposed by Wilke in 2008, which was a time-varying multivariate method aiming to estimate rapidly changing connectivity relationship of the brain [20]. Some studies have confirmed that ADTF is suitable for the analysis of short duration signals [19, 21]. Similarly, the signal **X**(t) could be described as:

9 $$ {\mathbf{X}}\left( {\text{t}} \right) = \sum\limits_{{{\text{n}} = 1}}^{\text{p}} {{\mathbf{A}}\left( {{\text{n}},{\text{t}}} \right){\mathbf{X}}({\text{t}} - {\text{n}}) + {\mathbf{E}}({\text{t}})} $$

where **X**(t) is a vector that changes with time, **A**(n, t) is the time-varying coefficient matrices. Other parameters are the same as their definitions in DTF. ADTF measure $\upgamma_{\text{ij}}^{2} \left( {\text{f}} \right)$ could be similarly defined as:

10 $$ \upgamma_{\text{ij}}^{2} \left( {\text{f}} \right) = \frac{{\left| {{\mathbf{H}}_{\text{ij}} \left( {{\text{f}},{\text{t}}} \right)} \right|^{2} }}{{\mathop \sum \nolimits_{{{\text{m}} = 1}}^{\text{k}} \left| {{\mathbf{H}}_{\text{im}} \left( {{\text{f}},{\text{t}}} \right)} \right|^{2} }} $$

Matlab toolbox eConnectome (Biomedical Functional Imaging and Neuroengineering Laboratory, University of Minnesota, Minneapolis, http://econnectome.umn.edu/) was used to compute the DTF and ADTF out-to-in of these signals. The out-to-in information indicates the causal information flows from one channel to another [22]. From out-to-in information we could compute the outflow information (or outflows) and inflow information (or inflows) of each flow. The outflow information of an electrode is the sum of the information from this electrode to all the other electrodes while its inflow information is the sum of all the other electrodes’ information flow into this electrode [22]. The outflow or inflow information value represents the ability of the electrode to affect other electrodes or to be affected from other electrodes. In this paper, the electrode with strong outflows was defined as a key cause node and the electrode with sensitive inflow information was considered as a key result node.

#### Surrogate data

Surrogate data, a time series which fits well with the linear-dynamics null hypothesis, could assess the significance of DTF and ADTF connectivity measures [19, 23]. It demonstrated that this method is suited for DTF and ADTF analysis that are the measurement of frequency-specific causal interactions [19]. The significance setting in this study was P < 0.05.

## Results

### Visual comfort comparison among different disparities measured by Stereo-VEPs

#### Waveforms and time–frequency analysis

**Fig. 3:**
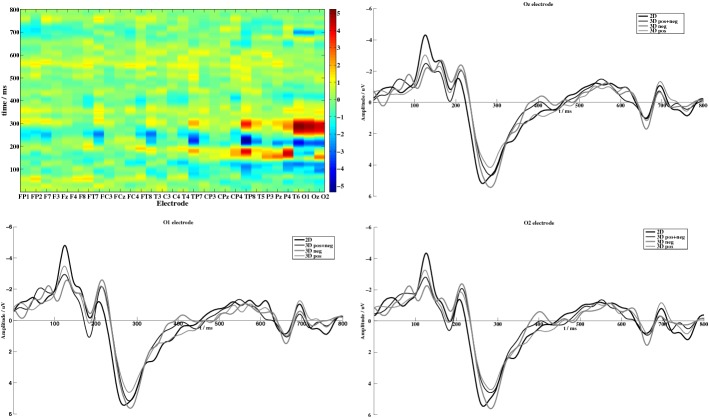
One representative planform of Stereo-VEP and the grand averages of Stereo-VEP at occipital electrodes

According to the subjective feedback, EEG signals in the cases of visual comfort were firstly averaged among subjects. Figure 3 depicts a representative planform of Stereo-VEP from one subject and averaged Stereo-VEP over all subject evoked by four types of stimuli at O1, Oz, O2 electrodes, respectively. In the planform, the magnitude of each amplitude was represented by colors. Colors from blue to red depict the amplitude of EEG signal from low to high. Obviously, the most distinct peak presented in the occipital lobe. In the grand average waves of Stereo-VEP, the P3 component was extremely obvious at nearly 270 ms after the onset of the stimulus (in the following contents, we use P270 for convenience). Table 1 lists the means and standard deviations of P270 from 10 participants. Compared with VEP evoked by ‘S1’, P270 evoked by other stimuli had a delay no less than 10 ms. As shown in Table 1, ‘S2’ caused nearly the same latencies at both the left and the right occipital lobe. In the case of ‘S3’, the latency of P270 at the left occipital lobe (O1) is slightly greater than that at the right part (O2), while ‘S4’ led to the contrary result. It is also quite clear that ‘S3’ evoked the most significant peak in the amplitude of P270.

**Table 1:** Means and standard deviations on the latency and peak amplitude of P270 over 10 subjects

Image	Latency (ms)			Peak (μV)		
	O1	Oz	O2	O1	Oz	O2
S1	269 ± 15	267 ± 12	271 ± 14	6.23 ± 1.93	6.02 ± 2.01	6.33 ± 2.86
S2	288 ± 21	289 ± 20	288 ± 22	5.84 ± 1.42	5.52 ± 1.67	5.52 ± 1.83
S3	284 ± 22	281 ± 21	281 ± 20	6.80 ± 1.60	6.77 ± 1.71	6.97 ± 2.05
S4	279 ± 20	285 ± 24	281 ± 21	5.24 ± 1.81	4.90 ± 1.90	5.15 ± 2.21

The Pearson correlation coefficient showed that peak amplitudes of P270 had a mid-relevance with the disparity (O1: Pearson correlation coefficient = − 0.474, P = 0.006 < 0.01; Oz: Pearson correlation coefficient = − 0.480, P = 0.005 < 0.01; O2: Pearson correlation coefficient = − 0.459, P = 0.008 < 0.01). The Paired *T* test between the occipital P270 from any two different types of visual stimuli was shown in Table 2. It is shown that the difference can be significantly observed by P270 component from O1, Oz, and O2 electrodes at the occipital lobe. Although between-group differences cannot be illustrated using latency and amplitude information from one single electrode, P270 based on the integrated results from three electrodes located at occipital lobe could be used as an effective indicator for differentiating different type of stimuli.

**Table 2:** The Paired T-test of P270

Pairs	Latency (ms)			Amplitude (μV)		
	O1	Oz	O2	O1	Oz	O2
S1–S2	T = − 4.862 P = 0.001	T = − 7.735 P = 0.000	T = − 4.235 P = 0.002	T = 3.110 P = 0.013		T = 3.233 P = 0.01
S1–S3	T = − 2.648 P = 0.027	T = − 2.776 P = 0.022				
S1–S4	T = − 2.454 P = 0.037	T = − 3.995 P = 0.003	T = − 2.669 P = 0.026		T = 4.125 P = 0.003	T = 4.302 P = 0.002
S2–S3					T = − 3.363 P = 0.008	T = 3.423 P = 0.008
S2–S4			T = 2.504 P = 0.034			
S3–S4				T = 3.324 P = 0.009	T = 4.703 P = 0.001	T = 3.887 P = 0.004

In addition, when the stimulation disappeared, an off-response at about 660 ms in the grand average showed that ‘S3’ caused the most significant changes in amplitude (Fig. 3). Previous study [24] suggested that off-responses had close relationship with visual persistence. It may infer that large crossed disparities would contribute to more off-responses. Another negative potential showed at around 700 ms, which could be due to the open issue of visual N700 [25, 26].

**Fig. 4:**
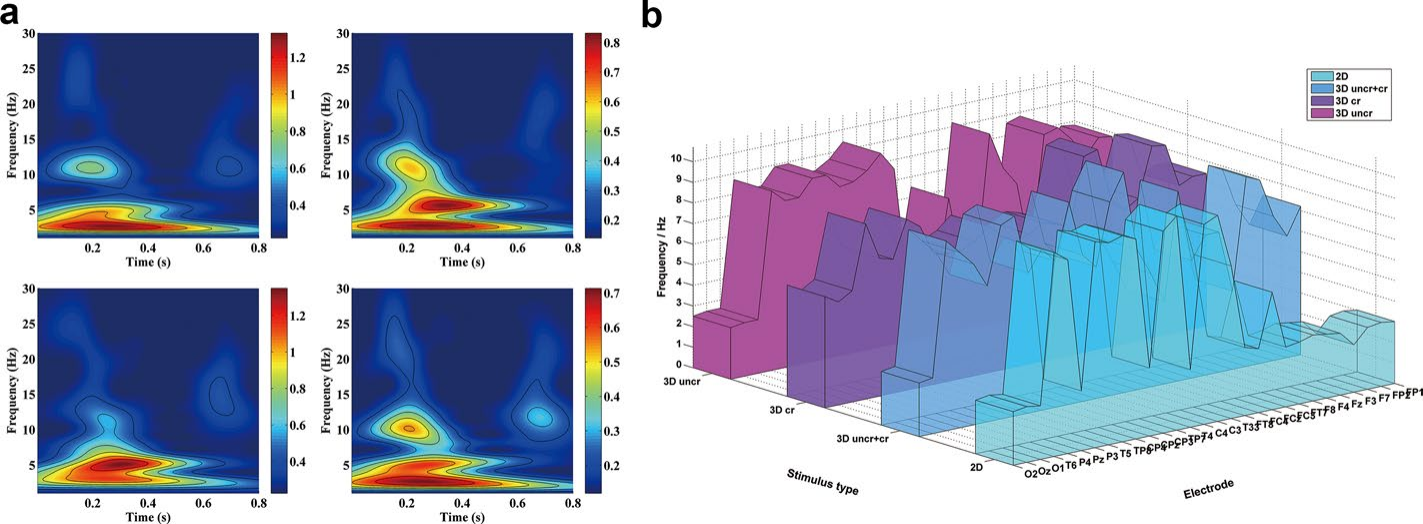
**a** Time–frequency map of averaged Stereo-VEP at Oz electrode upon visual stimuli with various disparities. Upper row in from left to right: TF-Analysis images upon stimuli with ‘S1’ (2D image) and ‘S2’ (3D image with both uncrossed and crossed disparities); Bottom row from left to right: TF-Analysis image upon stimuli with ‘S3’ (3D image with large crossed disparity) and ‘S4’ (3D image with large uncrossed disparity); **b** dominant frequency distributions of VEPs at P270 under stimulation with four different types of disparities

Figure 4a describes the time–frequency analysis (TF-Analysis) of averaged Stereo-VEP at Oz electrode within 800 ms after the onset of various visual stimuli. These images showed that P270 component evoked by 3D stereo stimuli includes wider frequency bandwidth (δ, θ and a few α bands) at occipital lobe as compared with the 2D stimulus. Figure 4b represents the dominant frequency distribution of P270, indicating that although the frequency band of Stereo-VEPs became wider upon 3D stereo stimuli, most of leading frequency were still at δ band (about 2.6 Hz). An exception was that the dominant frequency of Stereo-VEP evoked by ‘S3’ was 5.4 Hz, which was in θ band.

#### ADTF information flows

**Fig. 5:**
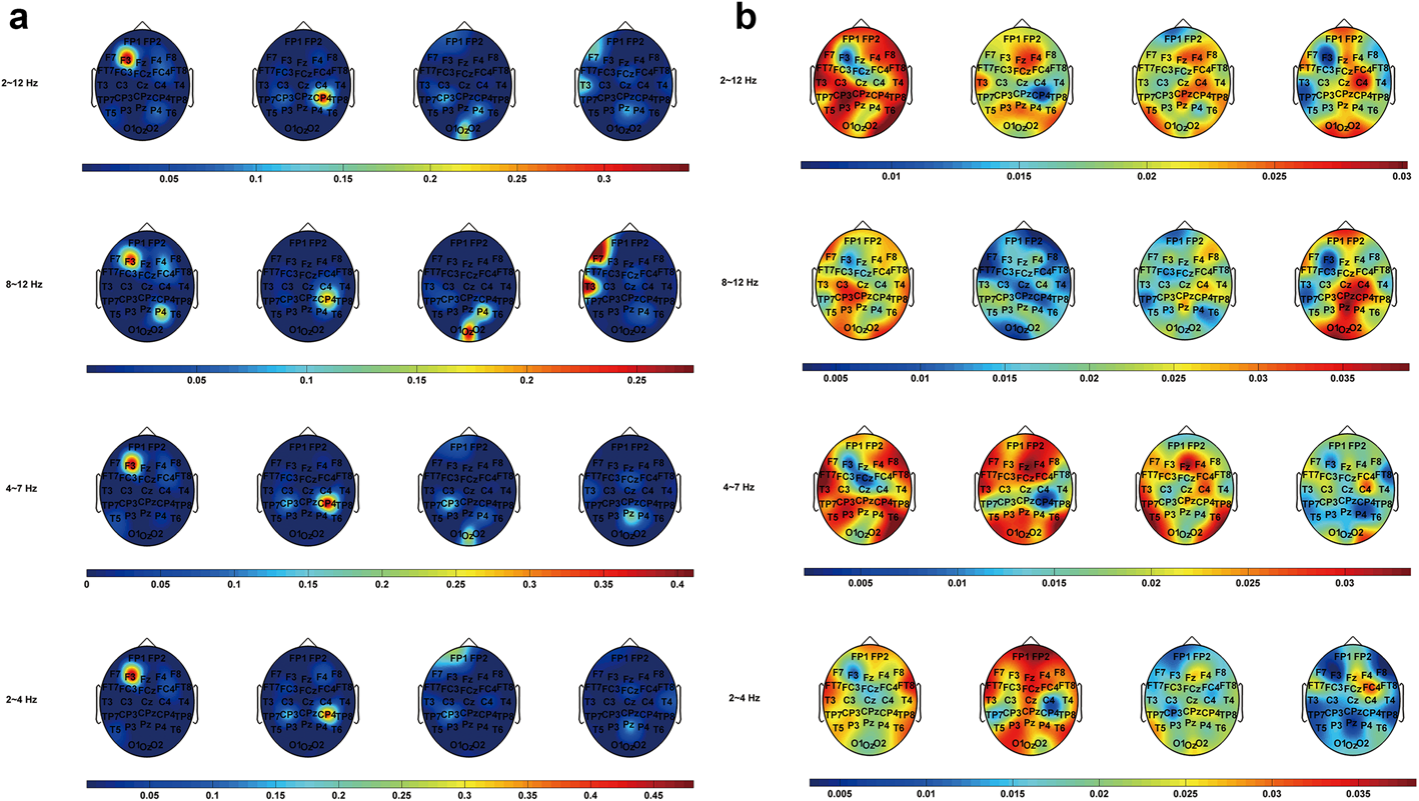
ADTF information flow topographies of P270 at different frequency bands (From top to bottom: α, θ, δ and δ ~ α (δ to α) band, from left to right: stimulation evoked by ‘S1’ to ‘S4’). **a** ADTF outflows, **b** ADTF inflows

δ, θ, α bands are related to signal matching and decision-making process, attentive processing and sensory processing, respectively [27]. Figure 5 shows the ADTF information flow topographies of P270 at δ, θ, α and δ ~ α (δ to α) bands (from bottom to top). In Fig. 5a, 2D stimulus evoked strong outflows at F3 electrode at δ ~ α bands, while 3D stimuli evoked that at F7, CP4, CP3, P4, Pz, T3 and Oz electrodes. It was easy to observe that 2D stimulus caused more powerful outflows in the left frontal lobe in the decision-making process (δ band), while 3D stimuli caused significant influence in the central lobe and the central-parietal lobe (CP4, CP3, Pz electrodes). In addition, ‘S3’ caused strong outflows at FP1 electrode as well. Although the θ band topographies were almost similar to the δ band topographies, Oz electrode at θ band became the key cause node instead of FP1 electrode in the δ band under ‘S3’ stimulation. In α band, P4 became one of the key cause nodes under ‘S1’ and ‘S3’ stimulation, while the CP3 electrode was never the key cause node under ‘S2’ or ‘S3’ stimulation. F7 and T3 were the key cause nodes under ‘S4’ stimulation. All the nodes mentioned above were marked as bright color in Fig. 5a. Generally, the ADTF outflows of P270 evoked by 2D stimulus mainly centered on the frontal lobe, while upon 3D stimuli the outflows of P270 centered on the posterior brain areas.

The ADTF inflow topographies of P270 were shown in Fig. 5b. The number of disparity types included in one stimulus was defined as depth complexity (D-complex). The D-complex of ‘S2’ was higher than that of ‘S3’ or ‘S4’ for the reason that it includes two types (uncrossed and crossed) of disparities. The result showed that the stimulus with high D-complex (‘S2’) caused stronger inflows at θ and δ bands (especially in the prefrontal area). Large crossed disparity evoked slightly stronger inflows when comparing the inflows between the two stimuli with low D-complex (‘S3’, ‘S4’). ‘S1’ and ‘S4’ caused relatively strong inflows in α band from the central area to the occipital lobe. The difference was that ‘S1’ acted on the left part while ‘S4’ acted on the right. ‘S3’ also slightly influenced the right frontal and central-parietal lobe at α band. The δ ~ α band topography for each stimulus showed that ‘S1’ owns the strongest inflows.

### Comparison between visual discomfort and comfort by DTF

#### Subjective feedbacks

**Fig. 6:**
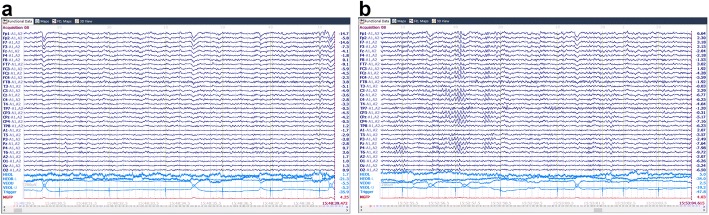
The contrast of the feedbacks during a session of the experiment. **a** At the beginning, **b** the point over half of the experimental time

The subjective feedbacks (Table 3) showed that no discomfort was reported during stimulation by ‘S1’, while ‘S3’ caused 93 feedbacks reporting discomfort. That is to say, observers are more sensitive to crossed disparity than to uncrossed ones, especially the large ones. This result is in good accordance with our previous study about stereoscopic depth in watching 3D films [28]. Furthermore, we found that most participants did not report feedbacks at the beginning of the experiment. As the experiment continues, discomfort feedbacks were frequently marked. Typically, as shown in Fig. 6a, there was no discomfort feedbacks (marked as ‘1’) recorded at the very beginning of the experimental session upon stimulation by ‘S3’ (marked as ‘30’), while discomfort feedbacks firstly appeared after half of the experimental process (Fig. 6b). Other labels ‘10’, ‘20’, ‘40’ in Fig. 6 represented ‘S1’, ‘S2’, and ‘S4’ respectively. The electrodes named A1 and A2 represented the references M1 and M2. It implied that, although disparity might easily lead to visual discomfort, there could still be an accumulative process.

**Table 3:** Subjective feedbacks

Image	Abbreviation	Feedback	Total number of stimuli
S1	2D	0	1200
S2	3D uncr+cr	39	1200
S3	3D cr	93	1200
S4	3D uncr	27	1200

### Comparison between visual discomfort and comfort by DTF

#### DTF information flows

**Fig. 7:**
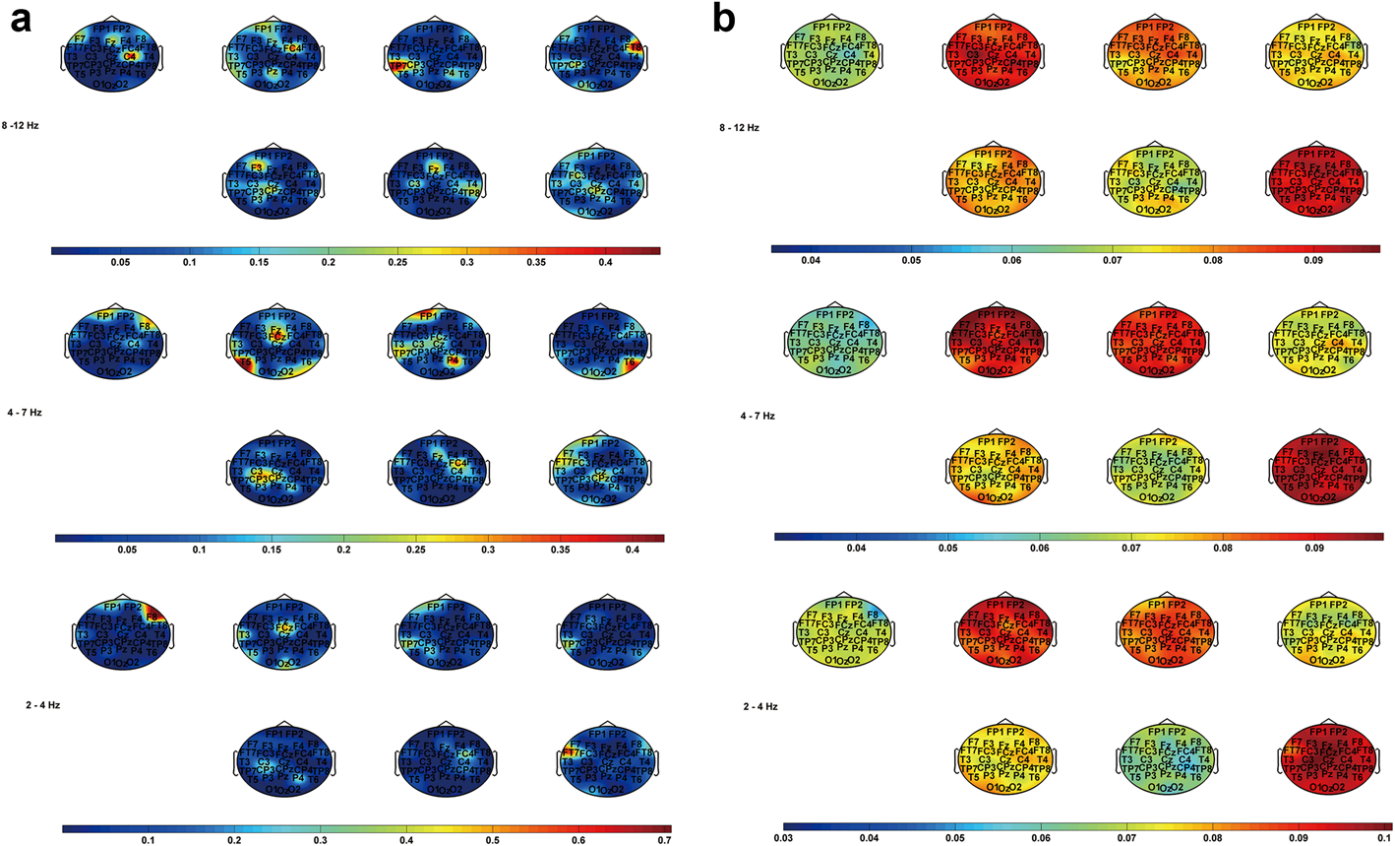
DTF information flow topographies at δ, θ, α bands (from top to bottom) under various stimulations (from left to right: ‘S1’ to ‘S4’). **a** DTF outflows, **b** DTF inflows. In the topography of each band, the images in the upper row represented the information flows with comfort report, while those in the bottom row were with discomfort report

Based on the subjective feedbacks, Stereo-VEPs were further divided into seven groups, dependent on the types of stimuli and subjective feelings (comfort and discomfort). Because in some cases uncomfortable feedbacks upon certain stimulation were rarely received, differences between comfort and discomfort at a specific time point could not be accurately represented. Therefore, the DTF method rather than the ADTF method was chosen to distinguish the difference between visual comfort and discomfort. Figure 7 depicted the DTF information flow topographies at α, θ, δ bands (from top to bottom). Data from 0 to 500 ms after the onset of the stimulus were analyzed. It was obvious that 3D stereo stimuli activated more and stronger outflows than the 2D stimulus did in posterior brain areas. In Fig. 7a, as compared to that in 2D condition, outflows became weaker in the right frontal lobe (F8 electrode) when participants received 3D stimuli no matter whether they reported visual discomfort or not. The comparison of comfort in this finding was in consistent with what has been illustrated in Fig. 5a. In ‘S2’, ‘S3’ and ‘S4’ cases where no discomfort was reported, the outflows in the left temporal lobe (T3 electrode) or in the left temporal-parietal lobe (TP7 electrode) became stronger, while the outflows in the same lobes became weaker if discomfort was marked in the feedback. The central lobe and the central-parietal lobe showed that the outflows at α and θ bands became relatively strong when participants received ‘S2’ and ‘S4’ stimuli and felt uncomfortable. However, the phenomenon was opposite under ‘S3’ stimulation.

As shown in Fig. 7b, the DTF inflows throughout the whole brain were obviously stronger under 3D stimulations than that under 2D stimulation. Consistent with Fig. 5b, the stimulus with high D-complex (‘S2’) caused stronger DTF inflows when participants felt comfortable. In terms of DTF inflows caused by low D-complex, compared ‘S3’ with ‘S4’, the large crossed disparity rather than uncrossed disparity evoked stronger DTF inflows in comfortable situation. It was also noticeable that the DTF inflows became weaker when subjects received ‘S2’ and ‘S3’ stimulation and felt uncomfortable, but the result was opposite in the ‘S4’ case.

## Discussion

Considering the safety issue, the largest disparity value used in this experiment was within the comfortable range proposed by Lambooij [1]. However, it is very close to the superior limit of the range and the effect was distinct when comparing with other stimuli in this paradigm. The uncomfortable feelings were frequently reported by participants when they received the stimulus with large crossed disparity.

### P270 and other Stereo-VEP components in stereo viewing

As VEP suggests the electrical activity of occipital lobe when the retina receives stimuli [17], VEPs in occipital region indicate that the change of disparity could influence the brain. In the overall averaged Stereo-VEPs, five onset response components C1, C2, C3, N2 and P3 were found. Early components of N75 component (or N1), P100 component (or P1) and N135 component (or N2) are often regarded as a set called the NPN component [29, 30]. Shawkat et al. verified the relevance between pattern reversal visual evoked potential (PRVEP) and POVEP, that is, P100 corresponds to C1, N135 corresponds to C2, and the following positive component may correspond to C3 [31, 32]. Sometimes, there is a small negative wave called C0, which corresponds to N75 [32]. In our study, it is shown in Fig. 3 that ‘S2’ and ‘S3’ caused distinct NPN component.

P1 (C1 in present study), N2 and P3 are regarded as neural correlate of visual consciousness and are usually used to study attention and visual processes [33]. Our results are consistent with previous studies which confirmed that C1 increases due to the depth perception [34, 35]. Compared to the 2D stimulus, a significant increase in the amplitude of C1 component in the 3D conditions was noticed. The difference in C1 amplitude between different stimuli can be explained by the difference between conditions, as the amplitude of C1 is known to be modulated by depth perception of 3D stimulus. As shown in Fig. 3, although ‘S3’ and ‘S2’ both evoked a clear C1 component at around 100 ms, more significant increases in C1 amplitude were found in ‘S3’ condition, which explains that the larger crossed disparity (‘S3’) may result in more distraction of visual attention and lead to more discomfort feedback. Although the result shown in Fig. 3 was the grand averaged amplitude of VEPs, there is no any statistically significance between different individuals participated in our experiment.

In order to attract participants’ attention and to avoid the decrease of attention intensity during stimulation, we used POVEP paradigm and forced participants to give feedbacks whenever there is any visual discomfort. P270 in this paper was P3b which appears in voluntary attention [36]. The TF-Analysis inferred that the P270 component at occipital lobe correlated closely to the decision-making process and attentive processing. As shown in Table 1, the latency of P270 appears to be longer in the case of ‘S2’, indicating that the visual cortex may need longer period of time for fusion and stereopsis upon processing images with increased disparity complexity. Similar to C1, the amplitude of P270 also increases with the depth perception by reflecting enhanced neural activity upon stimuli with larger disparity. Therefore, the occipital P270 could be chosen as an index to distinguish the effect of stereoscopic depth under comfortable feelings in stereo viewing.

The ADTF analysis showed that outflows of P270 caused by 2D stimulus were mainly on the frontal lobe, while 3D stereo stimuli evoked outflows of P270 on the posterior brain areas. It suggests that ADTF could help to judge whether the visual stimuli include any stereoscopic depth information under the comfortable state. Furthermore, the key cause nodes showed no significant change in ‘S1’ and ‘S2’ condition at δ, θ and α bands. However, in the large disparity cases, they changed obviously. According to the topographies of ADTF inflows, the result could be helpful to distinguish which kind of depth the stimulus may contain.

### Comparison between comfort and discomfort caused by stereoscopic depth

Researchers have demonstrated that the posterior parietal cortex participated actively in processing depth based on fMRI and functional near infrared spectroscopy (fNIRS) methods [37, 38]. In our study, changes of DTF and ADTF outflows were also found at the central-parietal lobe when stimulated by 3D images with different stereoscopic depth (Figs. 5 and 7). The key cause nodes appeared in posterior brain areas under 3D condition, while the nodes were in the frontal lobe under 2D condition. The subjective feedbacks indicated that ‘S3’ caused the most significant visual discomfort. DTF outflows in Fig. 7 shows that visual discomfort is accompanying with the decrease of the importance of the cause node in left temporal lobe. Based on literatures, temporal lobe relates to visual memory, language comprehension, and emotion association [39]. The ventral part of the temporal cortices also participates in high-level visual processing of complex stimuli. Anterior parts of the ventral stream are involved in object perception and recognition during visual processing [40]. Thus, it implies that discomfort from depth perception may diminish the connection strength between temporal lobe and other parts of the brain. On the other hand, the parietal lobe relates to attention and the central area is correlated with information processing. It was shown that the outflows at θ bands in the central lobe and the central-parietal lobe became relatively strong in participants under ‘S2’ and ‘S4’ stimulation accompanied with visual discomfort. However, the phenomenon evoked by ‘S3’ was opposite. It infers that the discomfort caused by the uncrossed disparity may incline to catch participants’ attention but that caused by the crossed disparity may reduce the attention. Figure 7b shows high depth complexity and large crossed disparity made DTF inflows weaker when participants felt uncomfortable, but it was contrary in the case with large uncrossed disparity. That is to say, the high depth complexity and the large crossed disparity would reduce the connectivity strength but the large uncrossed disparity would increase the connectivity strength when participants felt uncomfortable. This result indicated that DTF information flows from EEG signals could be considered as an index to distinguish the comfort level in stereo viewing.

Generally, this study proposed a Stereo-VEP experiment that can capture the fast responses upon short-term visual stimuli with various stereoscopic depth to minimize the interference from other factors. Compared to previous experiments, it focused more on the comfort level caused by disparity itself. ADTF and DTF results in this study illustrated that the information flows of EEG electrodes could be as effective as other methods to show the effect of disparities and comfort level. For the next step, based on the experimental and EEG analysis results in this paper, we intend to further explore the disparity-induced visual discomfort in stereo viewing in the cerebral cortex. Besides the findings reported herein, we have also found some strongly activated gyri at frontal and temporal lobes when viewers receiving stimuli with visual discomfort, which correlated well with previous studied using fMRI analysis.

## Conclusions

In this paper, we established a short-term experimental system which combined viewer-interactive subjective feedback towards better understanding of visual comfort or discomfort impacted by different stereoscopic depth. The results showed that the occipital P270 had a mid-relevance to the disparity of the stimuli and its ADTF showed the strongly activated areas when viewers are receiving stimulations with different disparities. DTF of P270 during the presence of the stimuli helped understanding the difference between comfort and discomfort stimulated by the same disparity. The change impacted along with the presence of discomfort could be found from the DTF outflows at the temporal and temporal-parietal lobes in δ band, central and central-parietal lobes in α and θ bands, and the inflows in these three bands. The subjective feedbacks showed the discomfort situations remained as a result of cumulative effect. Overall, the study provided a preferable experiment to observe the effects of disparity based on the Stereo-VEP experiment. Reconstruction of the information flow following a visual stimulus could be helpful to match the stereoscopic effect with viewers’ state (comfort or discomfort status) induced by disparity and provided an intuitive and easy-to-read result in a more convenient manner.

## Abbreviations

ADTF: adaptive directed transfer function
D-complex: the depth complexity
DTF: directed transfer function
ECG: electrocardiograph
EEG: electroencephalograph
EOG: electro-oculogram
fMRI: functional magnetic resonance imaging
fNIRS: functional near infrared spectroscopy
F-VEP: flash visual evoked potential
MVAR: multivariate autoregressive
NIRS: near infrared reflectance spectroscopy
POVEP: pattern onset/offset visual evoked potential
PRVEP: pattern reversal visual evoked potential
P-VEP: pattern visual evoked potential
RDS: random dot stereogram
SBC: Schwarz Bayesian Criterion
Stereo-POVEP: stereo pattern onset/offset visual evoked potential
Stereo-VEP: stereo visual evoked potential
TF-Analysis: time-frequency analysis
VEP: visual evoked potential
2D or S1: zero disparity, i.e. the image stimulus includes neither uncrossed disparities nor crossed disparities
3D uncr+cr or S2: the image stimulus includes both uncrossed and crossed disparities
3D cr or S3: the image stimulus only includes the crossed disparity
3D uncr or S4: the image stimulus only includes the uncrossed disparity
